# Upconverter loaded MoS_2_ counter electrode for broadband dye-sensitized solar cell applications

**DOI:** 10.3389/fchem.2024.1511600

**Published:** 2025-01-15

**Authors:** J. Kawya, M. Durairaj, S. Anandan, T. C. Sabari Girisun

**Affiliations:** ^1^ Nanophotonics Laboratory, Department of Physics, Bharathidasan University, Tiruchirappalli, India; ^2^ Nanomaterials and Solar Energy Conversion Laboratory, Department of Chemistry, National Institute of Technology, Tiruchirappalli, India

**Keywords:** MoS_2_ counter electrode, upconverter nanoparticles, DSSC, broadband absorption, upconversion (UC) materials

## Abstract

An interesting approach of including an upconverter in the MoS_2_ counter electrode can yield broadband light harvesting Pt-free DSSC assembly. Here different upconverter (UC) nanoparticles (Yb, Er incorporated NaYF_4_, YF_3_, CeO_2_ & Y_2_O_3_) were synthesized and loaded in MoS_2_ thin film by hydrothermal method. The inclusion of UCs in MoS_2_ films exposed without any secondary formation of upconverters and the uniform deposition of the films are confirmed through XRD and FESEM analysis respectively. The absorption spectrum (UV-Vis-NIR) confirms the increase in absorption intensity in visible and NIR regions due to the incorporation of UCs. Under 980 nm excitation, the UCs-loaded MoS_2_ films show emission in blue (450–490 nm), green (520–540 nm) and red (600–700 nm) regions due to the f-f inner transition occurring in the Er ions. The oxide and fluoride UCs-based DSSCs were fabricated with an FTO/TiO_2_/N719/Triiodide electrolyte/UC@MoS_2_/FTO assembly. Oxide UCs show greater electrocatalytic activity, which might be owed to the films exposed with more catalytic active sites favourable for ion transportation in the I^−^/I^−^_3_ electrolyte. Among them, higher photoconversion efficiency (PCE) of 7.1% was witnessed for (Y_2_O_3_: Er, Yb) @MoS_2_ counter electrode-based DSSC which is due to the good conductivity (R_S_) of the film, the longer electron lifetime and photon harvesting enhancement by upconversion process. It is notably convinced that the UC-loaded MoS_2_ films can be used as an effective counter electrode for the possible realization of upconverter DSSC and used for broadband light absorption.

## 1 Introduction

Global energy need and the equivalent sustainable power generation are on the constant rise to deal with the demand for energy for long-term practice. The production of adequate energy employing photovoltaic (PV) technology, i.e., solar cells signifies an assuring way for green and renewable energy generation. However, another prime cause that constrains the photo conversion efficiency (PCE) of solar cells is the spectral disparity between the photon energy of sunlight and the bandgap of the solar cell (Tadge et al., 2020; El Assyry et al., 2022; Tohluebaji et al., 2024). Another constraint of making trade-off between PCE and cost is although met through dye sensitized solar cells (DSSCs), low-priced PV technology is far from reality due to the expensive Pt based counter electrodes (Sarkar and Bera, 2020). Thus, the mutual outcome of having low-cost and high-efficiency Pt-free DSSCs might suggest an evolution in PV manufacturing. Recently, there has been growing interest worldwide in the development of electrocatalyst materials as potential counter electrode materials as it exhibits high electrical conductivity, excellent chemical stability, high mechanical strength and demonstrate superior catalytic activity (Hussain et al., 2015; Kanjana et al., 2023; Pujari et al., 2017). Among them, Platinum (Pt) is the most promising material for counter electrodes in DSSCs as they have outstanding charge carrier mobility and good electrocatalytic activity but is limited by its very expensive nature (Fang et al., 2004; Vikraman et al., 2018; Younas et al., 2020). Thus, cost-effective, environmentally and chemically long-standing Pt free CE such as inorganic compounds (PtAu, PtCo, FeCo_2_), conducting polymers (PANi, PPy, PEDOT), transition metal compounds including nitrides (VN, TiN, MoN), carbides (TiC, VC, Nb C), phosphides (CoP, Fe_2_P, Ni_12_P_5_), carbon constituents (CNT, carbon black, carbon fibre) to substitute pricy Platinum electrode in DSSCs have been recently explored among different scientific groups (Yu et al., 2018; Jeong et al., 2019). The plentiful and low-cost transition-metal dichalcogenides (TMDCs), a group of two-dimensional atomically thin layered materials which have the form of MX_2_ where M refers to transition metals and X refers to dichalcogenide elements (S, Se, Te) which validate analogous electrocatalytic behaviour of Platinum that intuitively allow them to be suitable candidates for counter electrodes in DSSC. Amid them, MoS_2_ (molybdenum disulphide), a typical van der Waals force stacked layered material consists of single layer of Mo packed between two layers of S. (Wei et al., 2016; Vikraman et al., 2019). Existing research on MoS_2_ proved that sulphur sites are responsible for the electrocatalytic activity of MoS_2_, which is suitable for photoelectrochemical applications. Recently Durairaj et al., reported that MoS_2_ nanoplatelets prepared by the hydrothermal method exhibit high electrocatalytic activity and comparable photo conversion efficiency (PCE) with Pt-based DSSC (η = 6.39%) (Sabari Girisun et al., 2023). Thus, MoS_2_ can be an excellent CE material for the fabrication of Pt-free DSSCs provided the photoconversion efficiency can be improved by certain means.

In this line, PCE of DSSCs is sturdily reliant on the competence of light-harvesting dyes. But commonly used ruthenium-based dyes possess an optical bandgap of 1.8 eV, which limits its photon absorbing capacity to a quite narrow spectral wavelength range between 300 and 700 nm (Shan, 2010). Thus, the prime constraint of improving PCE of DSSC is its inability to utilize solar radiation in the infrared spectrum region, which comprises almost half the energy (49.4%) of the sunlight. One inimitable methodology to subjugate infrared photons is the photon upconversion approach (UC) (Dutta, 2021). Rare-earth doped nanoparticles are guest–host systems actively used as photon upconverters in which trivalent lanthanide ions are incorporated in a suitable host frame. The forbidden quality of 4f–4f transitions in lanthanides produces intermediate energy levels of very long lifetimes (up to tens of a millisecond), thus supporting the successive excitations (low energy photons) in the excited states of one lanthanide ion (sensitizers) as well as granting more eligible ion-ion interactions in the excited states which cause energy transference between two or more lanthanide ions (emitters) that radiate higher energy photons. Among the available lanthanide ions, Ytterbium (Yb) and Erbium (Er) are identified as excellent sensitizer and emitter for UC mechanism (Yao et al., 2016).

In 2017, Wenwu Liu et al., synthesized Nb_2_O_5_-covered TiO_2_ nanowires doped with Er^3+^/Yb^3+^ as photoanode and attained a high efficiency of 8.2% as an effect of making fermi levels in the TiO_2_ (Lu et al., 2019). Dutta et al. (2019) reported an enhanced PCE that proves the add-on of the Y_2_O_3_: Ho^3+^/Yb^3+^ UC nanophosphors into the TiO_2_ film can effectively outdo the photovoltaic performance. Although several attempts so far have been made to incorporate UC in photoanode, it is limited by the restriction in reducing the conductivity, transparency and dye adsorption on the semiconductor metal oxide layer. Another way to improve the PCE of DSSC without altering electrode work functions is to incorporate UC in the counter electrode. The legitimate selection of the host material also plays an important role in obtaining high UC luminescence, as it influences the UC process through either the phonon dynamics or the local crystal field occurrence around the rare-earth ions (Ambapuram et al., 2020a). So far examined host materials for UC are oxides, fluorides, vanadates, oxysulphides and oxyfluorides. In this series, Ceria (CeO_2_) and Yttria (Y_2_O_3_) as oxide host materials and Sodium Yttrium Fluoride (NaYF_4_) and Yttrium Fluoride (YF_3_) as fluoride host materials can be an interesting upconversion process in DSSCs since they have been well explored in upconverter loaded photoanode based DSSCs (Yao et al., 2016; Durairaj and Sabari Girisun, 2023). Thus, exploring the effect of the incorporation of UCs in the counter electrode of DSSC, will provide a deep understanding of the role and suitable position of UCs in DSSCs. The present work UC-loaded MoS_2_ counter electrode based DSSC can pave the way towards the realization of broadband Pt-free DSSCs. A straightforward hydrothermal technique has opted to produce four UC-incorporated MoS_2_ counter electrodes namely CeO_2_/NaYF_4_/YF_3_/Y_2_O_3_: Yb, Er and analyse the performance of different broadband Pt free DSSCs (Durairaj and Sabari Girisun, 2023).

## 2 Experimental section

### 2.1 Materials

High purity (Sigma-Aldrich, United States) Thiourea (CH_4_N_2_S), Ammonium molybdate tetrahydrate (NH_4_)_6_Mo_7_O_24_.4H_2_O), Cerium nitrate [Ce (NO_3_)_2_], Sodium fluoride (NaF), Erbium chloride hexahydrate (ErCl_3_), Ytterbium chloride hexahydrate (YbCl_3_), Yttrium chloride hexahydrate (YCl_3_), Ammonium fluoride (NH_4_F), Poly Vinyl Alcohol [PVA- (C_2_H_4_O)n] and Fluoride Tin Oxide substrate (FTO) were purchased and used without any prior purification.

### 2.2 Synthesis of upconverter nanophosphors

The simple sol-gel procedure is employed to synthesise the oxide (CeO_2_: Yb, Er & Y_2_O_3_: Yb, Er) UCs. The precursor solution for CeO_2_: Yb, Er was prepared by dissolving 1 g of Cerium nitrate, 0.04 g of Erbium chloride, and 0.36 g of Ytterbium chloride in 10 mL of deionized water through constant stirring for 1 h. Then 1 g of PVA was added into the stirring solution and allowed for an extra 1 h stirring. Next, the precursor solution was transferred into the crucible and placed in the oven at 120°C for 12 h. Thus, the obtained pale-yellow colour powder was further annealed (600°C, 3 h). The same procedure was followed for Y_2_O_3_: Yb, Er by using yttrium chloride hexahydrate as the host precursor and yellow colour powder was obtained.

The hydrothermal method is used to synthesise the fluoride (NaYF_4_: Yb, Er & YF_3_: Yb, Er) UCs. The precursor solution for YF_3_: Yb, Er was prepared by taking 0.8 g of ammonium fluoride, 2.6 g of yttrium chloride, 0.36 g of ytterbium chloride and 0.04 g of erbium chloride in 50 mL DI water and after 1 h continuous stirring the solution was poured into a 75 mL Teflon-lined autoclave. The solution taken in 75 mL Teflon-lined autoclave was heated (180°C, 24 h) and the collected UC powder was further washed using DI water. Finally, the obtained UCs were dried (120°C, 12 h). The same procedure was followed for NaYF_4_: Yb, Er by using sodium fluoride as the host precursor and white colour powder was obtained.

### 2.3 Preparation of upconverter loaded MoS_2_ thin film as counter electrode

**FIGURE 1:**
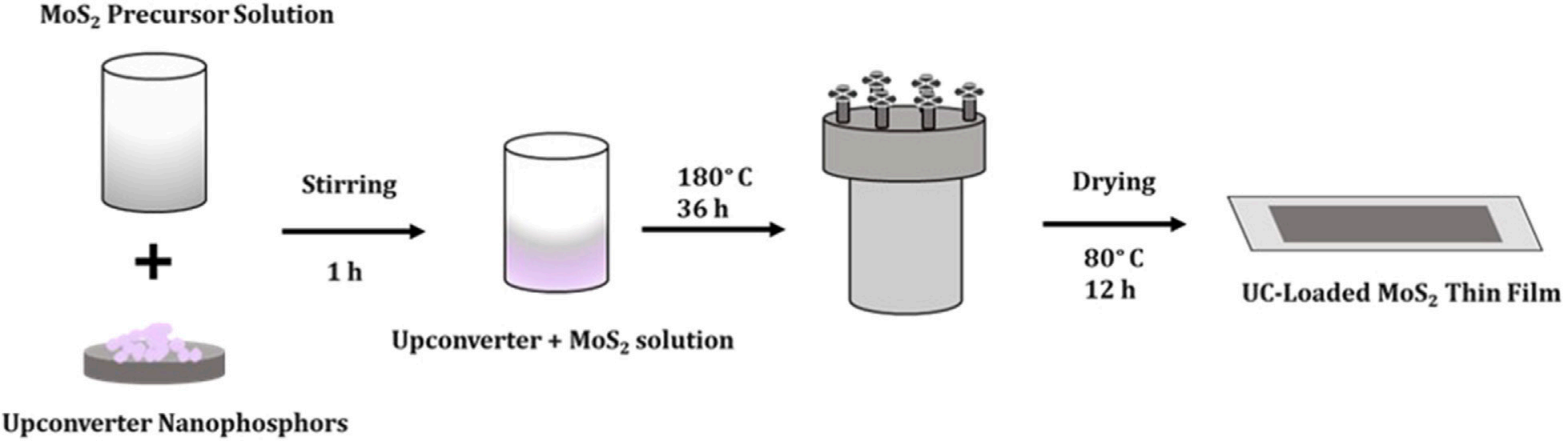
Preparation UC-loaded MoS_2_ CEs.

Initially the FTO substrates were cleaned by ultrasonication using a solution containing acetone, DD water and isopropanol taken in a 1:1:1 ratio. The MoS_2_ precursor solution was prepared by dissolving 1 g of ammonium molybdate tetrahydrate and 2 g of thiourea in 50 mL of DI water through constant stirring for 1 h at 70°C. As-prepared CeO_2_: Yb, Er weighing 0.02 g were separately sonicated in 10 mL DI water for 30 min. Then, the sonicated UC solution was poured into MoS_2_ precursor solution and the stirring was continued for another 1 h without heat treatment. The pre-cleaned FTO plates (2 × 2 cm^2^) were placed at 45° angle against the wall of the Teflon container. Next, the solution was heated treated (180°C, 36 h) in a steel autoclave to obtain UC-loaded MoS_2_ thin films. Finally the DI water washed films were dried at 80°C for 12 h to achieve crystalline CEs (see in Figure 1). The same procedure was followed for other UCs and samples were labelled as CM (CeO_2_: Yb, Er), YOM (Y_2_O_3_: Yb, Er), NM (NaYF_4_: Yb, Er), and YM (YF_3_: Yb, Er).

### 2.4 Fabrication of DSSCs

**FIGURE 2:**
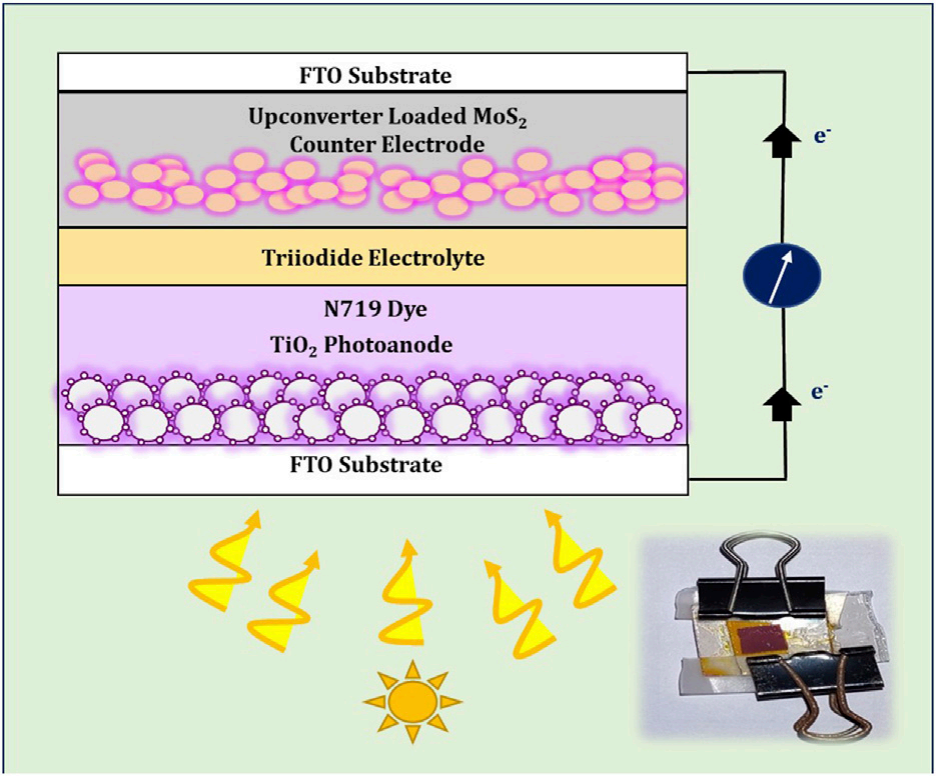
Layout of DSSC- assembled DSSC (insert).

The layout of the constructed DSSC and real-time image of the assembled DSSC are shown in Figure 2. The doctor-blade method was used to prepare mesoporous TiO_2_ photoanodes on an FTO substrate and the film was sintered at 450°C for 1 h. The prepared photoanodes were immersed for 24 h in 0.5 mM of N719 dye solution containing acetonitrile and tert-butanol taken in a 1:1 ratio. The DSSCs were fabricated by coupling the dye-sensitized TiO_2_ photoanodes and the UC incorporated MoS_2_ counter electrodes concurrently and the iodine electrolyte (containing Iodine, Lithium Iodide, 4-tert-butylpyridine and acetonitrile) was inoculated between the photoanode and counter electrode.

### 2.5 Characterization and measurements of counter electrodes and DSSCs

The X-ray diffraction analysis was done by an Empyrean instrument with Cu-Kα (λ = 1.54 Å) for the prepared UC-loaded MoS_2_ counter electrodes for crystallization and structural evaluation. Field emission scanning electron microscopy (FESEM) imaging by ZEISS Sigma instrument was used to examine the surface morphology of the films. The absorption properties of the prepared thin films were studied by UV-Vis-NIR spectrometer of wavelength from 175 nm to 3,300 nm (Shimadzu 3600). The emission features of the samples were analysed by a photoluminescence spectrometer. The fabricated DSSCs were illuminated by the solar simulator (150 W, Oriel) and their corresponding I-V characteristics were recorded. Enlitech’s IPCE spectral instrument was utilized to study the PCE of the DSSCs. Electrochemical impedance spectra in 1–106 MHz frequency range were obtained using a solar simulator and a potentiostat with an impedance analyser (AUTO-LAB12/FRA2).

## 3 Result and discussion

### 3.1 Phase and morphology of UC-loaded MoS_2_ films

**FIGURE 3:**
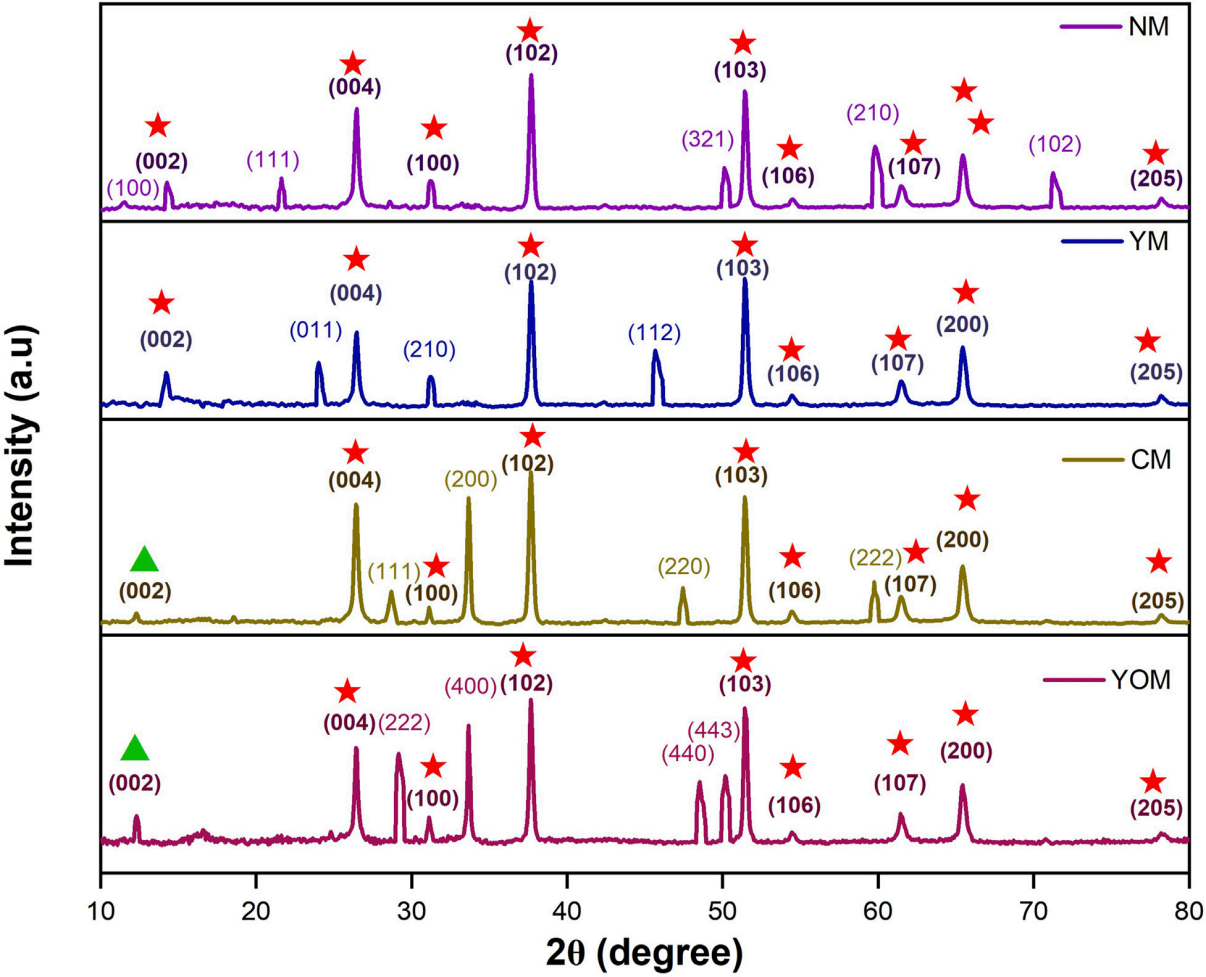
X-Ray Diffraction Pattern for UC-loaded MoS_2_ Thin films (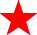-2H phase of MoS_2_ & 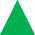
- 1T phase of MoS_2_).

XRD was used to analyse the structure and phase of UC-loaded MoS_2_ thin films. As shown in Figure 3, the indexed XRD peaks signify the existence of both MoS_2_ and upconverter nanoparticles validating the formation and deposition of nanocomposites in the films. Here in the nanocomposite thin films, MoS_2_ exist in 1T-2H mixed-phase in oxide UCs and 2H phase in fluoride UCs [JCPDS Card No. (65-1951)]. Further the presence of upconverter nanoparticles NaYF_4_: (Yb, Er) in hexagonal phase [JCPDS Card No. 16-0334], YF_3_: (Yb, Er) in orthorhombic phase [JCPDS Card No. 74-0911], CeO_2_: (Yb, Er) in cubic phase [JCPDS Card No. 04-0593] and Y_2_O_3_: (Yb, Er) in cubic phase [JCPDS Card No. 25-1200] is also well accorded. The Yb and Er lanthanide ions are completely incorporated into the host lattices of upconverters (NaYF_4_, YF_3_, CeO_2_, Y_2_O_3_) in the lattice site of Y and Ce appropriately, which justifies the non-emergence of any impurity segments of upconverters which proved the phase purity of nanocomposites (Ambapuram et al., 2020a; Durairaj and Sabari Girisun, 2023). In Figure 3, the (002), (004), (100), (102), (103), (106), (107), (200) and (205) planes refer to the 2H-hexagonal phase which is a semiconductor phase of MoS_2_. The (002) plane of 2H MoS_2_ located at 14.2° is identified as the basal plane, which exists in NM and YM films. Also, the CM and YOM films display a 1T-2H mixed phase of MoS_2_ which is identified by a lower peak shift at 11.2° that corresponds to the (002) basal plane of the 1T phase which is a metallic phase of MoS_2_ (Sabari Girisun et al., 2023). The (002) plane of MoS_2_ specified as the basal plane arises due to interlayer scattering of Mo–Mo and this plane appears with low intensity in all the films which shows that the MoS_2_ nanograins and upconverter nanoparticles were grown upon the MoS_2_ nanosheets during the hydrothermal process, consequently resulting in the poor crystallinity due to the lacking of the layered structure of MoS_2_ (Vikraman et al., 2018; Ghaleghafi et al., 2021). The active centres present in the basal plane of the 2H phase (14.2°) show low conductivity when compared to the edge plane active centres whereas the basal plane of the 1T phase (11.2°) is as catalytically active as edge planes (Das and Parida, 2021). Thus, the mixed 1T-2H phase MoS_2_ in CM and YOM films facilitate the fast electron transportation during the electrocatalytic reaction which can enable high current density in oxide UCs-loaded MoS_2_ CE-based DSSCs.

**FIGURE 4:**
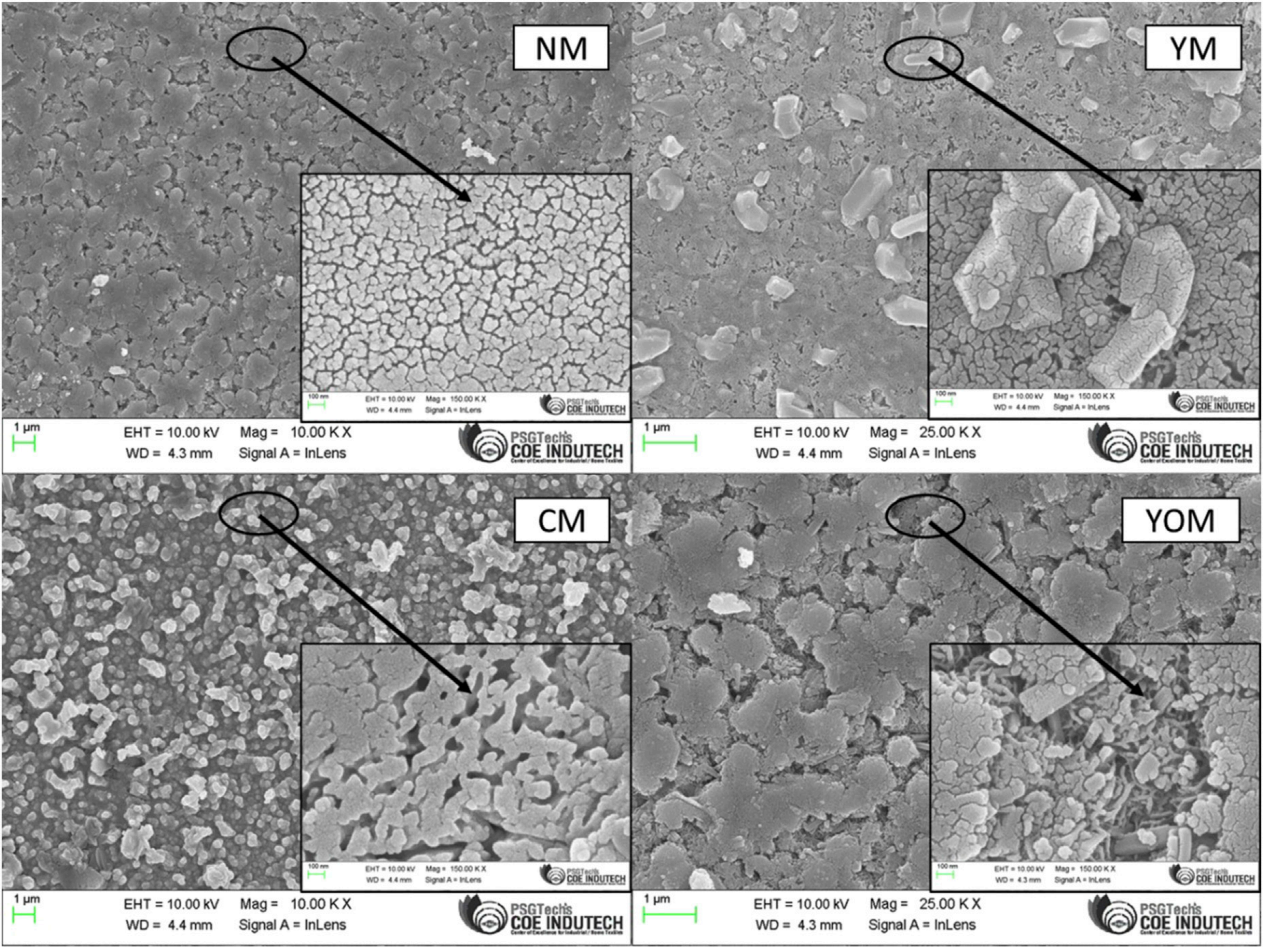
Surface Morphology for UC-loaded MoS_2_ Thin films.

The surface morphology of UCs-loaded MoS_2_ films is shown in Figure 4 and the hydrothermal method of producing films reveals aggregated morphology with the uniform and continuous distribution of dissimilar small grains in all the films with an average particle size of 40–50 nm. The existence of UCs such as CeO_2_: (Yb, Er), Y_2_O_3_: (Yb, Er), NaYF_4_: (Yb, Er) and YF_3_: (Yb, Er) has a less significant impact on the nanograin morphology of MoS_2_ which is mainly due to the incorporation of low concentration of UCs but greatly induce the formation of porosity in the films. These MoS_2_ nanograins are linked to form bonded clusters throughout the films. These fused clusters commence the porosity and voids during the growth of the MoS_2_ films on the FTO substrate (Pujari et al., 2017). This porous grouping is observed in all the films and the distribution of MoS_2_ nanograins is associated with each other which enables good electron transportation in electrochemical performance (Gopakumar et al., 2019). MoS_2_ nanograins congregated all over the MoS_2_ nanosheets which is confirmed by the presence of lattice orientation for the layered structure of MoS_2_ indicated by the observed conservative plane of (002). Furtherly, the porous appearance of UCs-loaded MoS_2_ films would provide a large specific area having large reduction sites to subjugate high electrocatalytic activity that promotes the I^−^/I^3−^ redox reaction which constitutes a better electrolyte-counter electrode interface and hence can improve the electrocatalytic activity (Kanjana et al., 2023).

### 3.2 Absorption properties of UC-loaded MoS_2_ films

**FIGURE 5:**
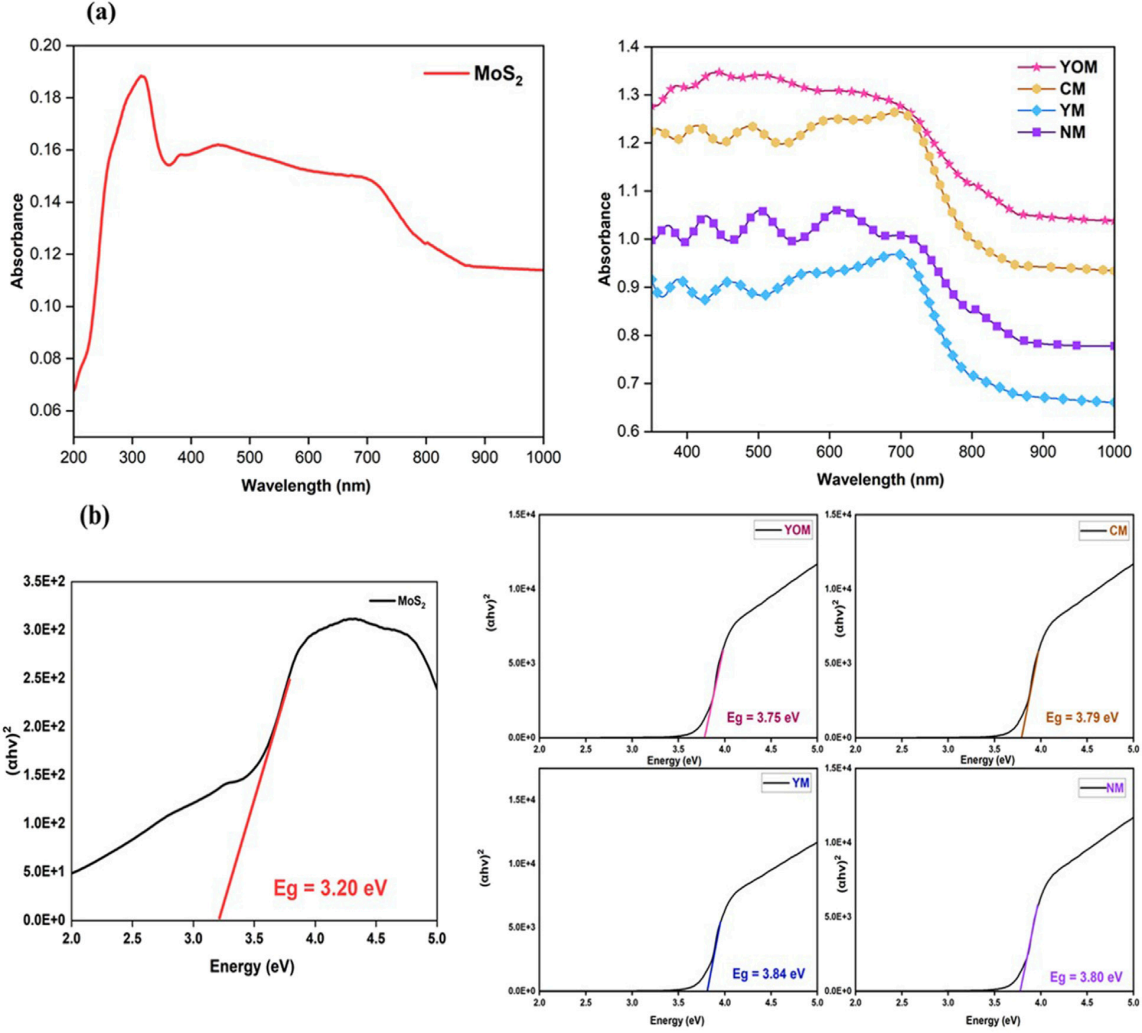
**(A)** Absorption spectra of MoS_2_ and UC-loaded MoS_2_ CEs 5. **(B)** Tauc plot of MoS_2_ and UC-loaded MoS_2_ CEs.

The optical responses and Tauc’s plots of the pure MoS_2_ and UC-loaded MoS_2_ films are shown in Figures 5A, B. From the absorption spectra, UC-loaded MoS_2_ films disclose enhanced absorption behaviour in the visible region than pure MoS_2_. The thin films show considerable absorbance featuring in the NIR spectral range up to 1,200 nm due to the incorporation of UCs, whereas the absorbance is intensified in the UV and visible regions, which is effectively heightened by the upconversion process facilitated by Er and Yb ions in the CEs during the illumination (Gopakumar et al., 2019). The absorption spectrum displays absorption bands around UV (350–400 nm) and visible regions (450–550 nm) due to the electron-transfer activity coupled with extrinsic states of MoS_2_ such as the presence of defect states and oxygen vacancies developed because of the inclusion of UCs which created more sub-band gaps between them (Ghaleghafi et al., 2021). The absorption over the 600–800 nm region corresponds to the transition from the k-point of the Brillouin zone of MoS_2_ and the edge observed at 390 nm and 420 nm can be consigned to the MoS_2_ charge transition from the deep valence band to the conduction band (Hussain et al., 2015). The optical bandgap of the UCs-loaded MoS_2_ thin films was determined to be 3.80 eV (NM), 3.84 eV (YM), 3.79 eV (CM) and 3.75 eV (YOM) respectively by Tauc plot shown in Figure 5B. The incorporation of UCs altered the band configuration of MoS_2_ and the oxide UCs loaded MoS_2_ thin films show lower bandgap compared to the fluoride UCs loaded MoS_2_ thin films due to the presence of oxygen vacancies which facilitates electron conductivity in the film (Sabari Girisun et al., 2023). The outcomes confirm the insertion of upconverter nanoparticles in DSSCs can stimulate the large visible light absorption ability of N719 dye (400–700 nm), which guarantees an opening for the harvesting of NIR light, a major component of sunlight. Here the oxide UCs, CM, and YOM exhibit higher absorption intensity than fluoride UCs due to the high chemical and optical stability of oxide UCs which helps in improving the photovoltaic performance whereas the fluorides are often hygroscopic, which have a limitation towards stability.

### 3.3 Photoluminescence and upconversion properties of UC-loaded MoS_2_ films

**FIGURE 6:**
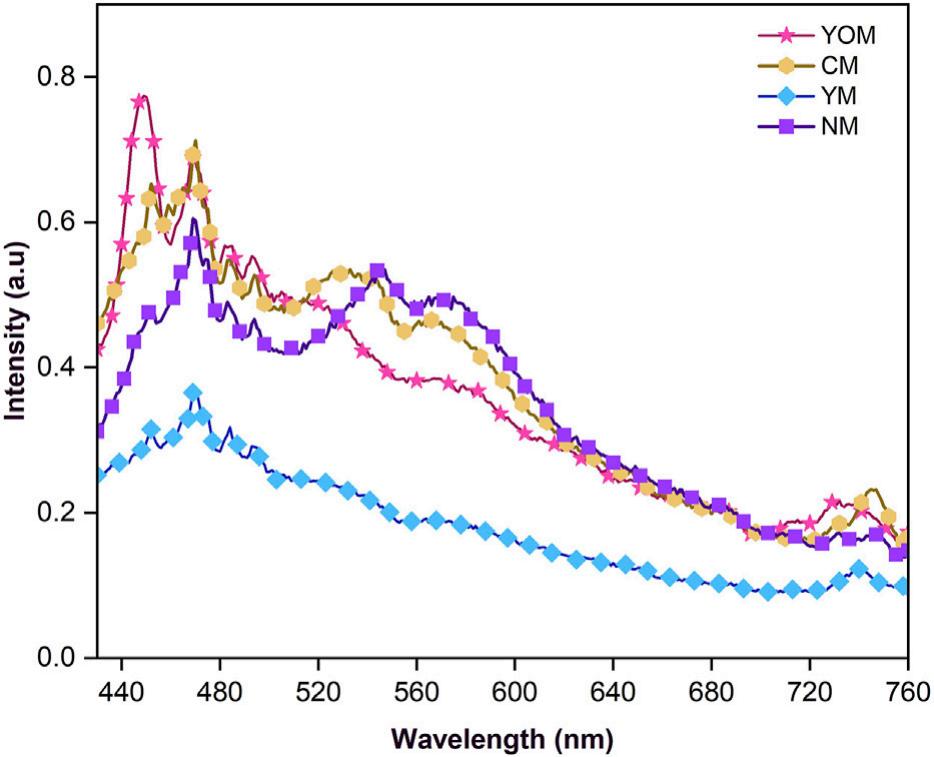
Emission spectra for UC-loaded MoS_2_ CEs.

**FIGURE 7:**
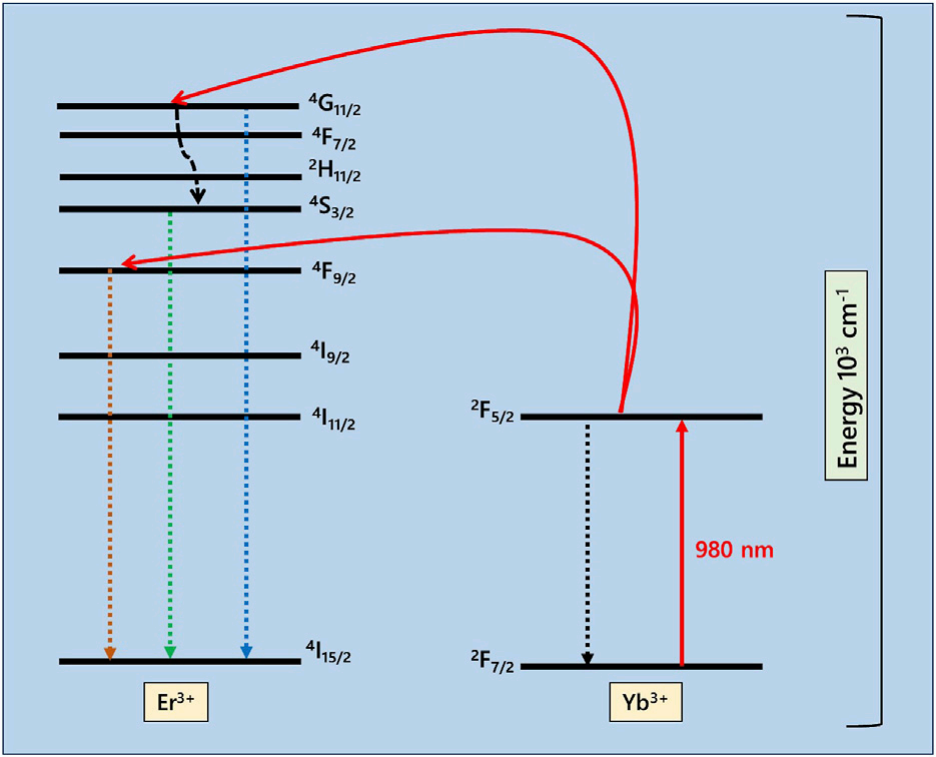
Upconversion luminescence process.

**FIGURE 8:**
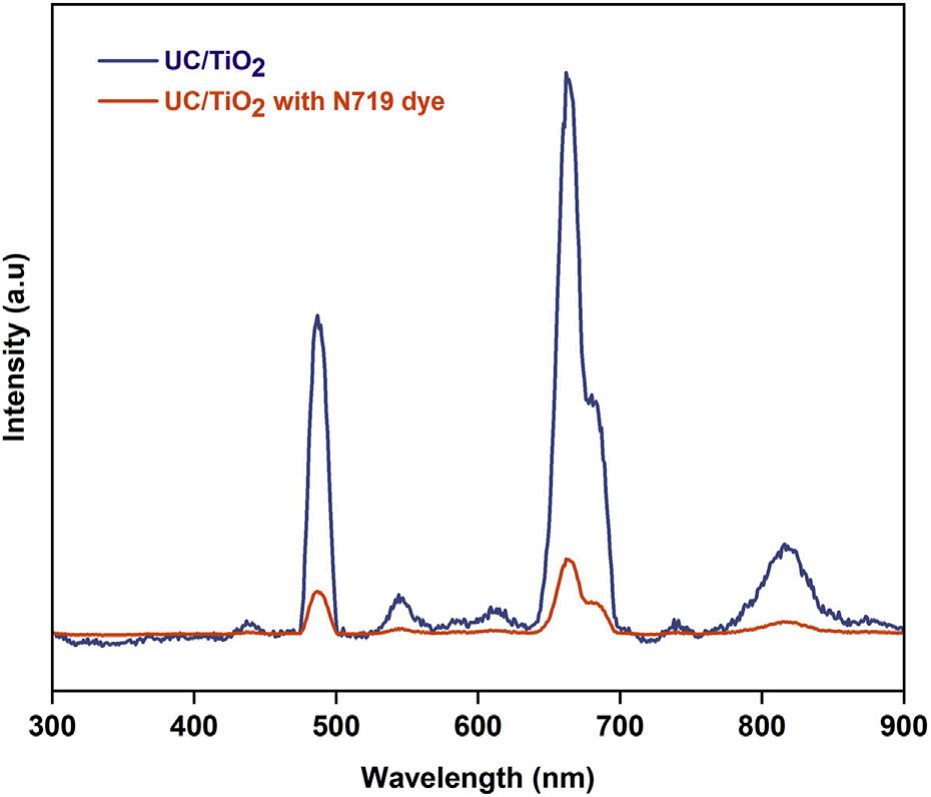
Luminescence spectrum of UC-loaded TiO_2_ thin film with and without N719 sensitization.

The emission spectrum of the prepared UC-loaded MoS_2_ thin films is shown in Figure 6. Under 980 nm excitation, the photoluminescence spectrum demonstrates strong blue (440–490 nm), green (520–540 nm) and weak red emissions (600–700 nm) which are attributed to ^2^H_11/2_, ^4^S_3/2_ → ^4^I_15/2_, ^4^F_9/2_ → ^4^I_15/2_ and ^4^I_13/2_ → ^4^I_15/2_ f-f transitions of emitter (Er^3+^) ions respectively (Li et al., 2014; Zhao et al., 2014). The emission peaks that occur at the visible region (570–580 nm) correspond to the surface states of MoS_2_ and also the peaks around 740 nm correspond to the valence band splitting caused by the strong spin-orbit coupling of MoS_2_ (Vikraman et al., 2018; Wilcoxon and Samara, 1996). Notably, the emission peaks are just falling in the absorption range of N719 dye, which has strong absorption in the visible range (400–700 nm). Here, the oxide UCs (CM, and YOM) deliver better emission intensity in the dye-absorbing region. The suppressed emission noticed for fluoride UCs is due to the happening of multiphonon relaxation in the meta-stable states as they have lower phonon energies. To understand the upconversion luminescence, the energy transfer mechanism is shown in Figure 7. The Yb^3+^ ions present in the UCs are excited from ^2^F_7/2_ energy (ground) state to the ^2^F_5/2_ energy (higher) state which has a large absorption coefficient in that state and they go back to the ground state by radiating the energy. The radiated energy from the sensitizer Yb^3+^ ions is transferred to various energy states of emitter Er^3+^ ions, which have ladder-like energy levels, resulting in the ^4^F_9/2_ and ^4^G_11/2_ levels. After undergoing a multiphonon relaxation, a fraction of the ^4^G_11/2_ level decays to the ^4^S_3/2_ level. Meantime, another photon is absorbed by Yb^3+^ ions and the energy is transferred one more time to the emitter Er^3+^ ions. Thus, the ^4^F_9/2_ and ^4^G_11/2_ energy states are populated in Er^3+^. Afterwards, the excited electrons undertake non-radiative processes and decay to the ground state of Er (^4^I_15/2_) (Dutta, 2021; Yao et al., 2016). Therefore, visible emissions are observed owing to this f-f transition of UCs. Thus, the emission studies clearly emphasize that the IR photons from the sunlight can be reabsorbed by the dye N719 in the form of visible photons by upconversion phenomenon and the absorption potential of N719 dye can be prolonged, consequently the performance of the DSSC can be improved. To witness this, the UC-loaded TiO_2_ film (Y_2_O_3_:Yb, Er) was prepared and sensitized with N719 dye and both the films with and without N719 dye sensitization were subjected to 980 nm excitation and the upconversion emission intensity was recorded. The emission intensity of N719-sensitized UC-loaded TiO_2_ film reduces as the dye has high absorbance in the visible region as shown in Figure 8. This evidences the visible emission from the upconverter nanophosphors are reabsorbed by the dye, hence the DSSC’s efficiency is enhanced.

### 3.4 Electrochemical analysis of UC-loaded MoS_2_ CEs-based DSSC

**FIGURE 9:**
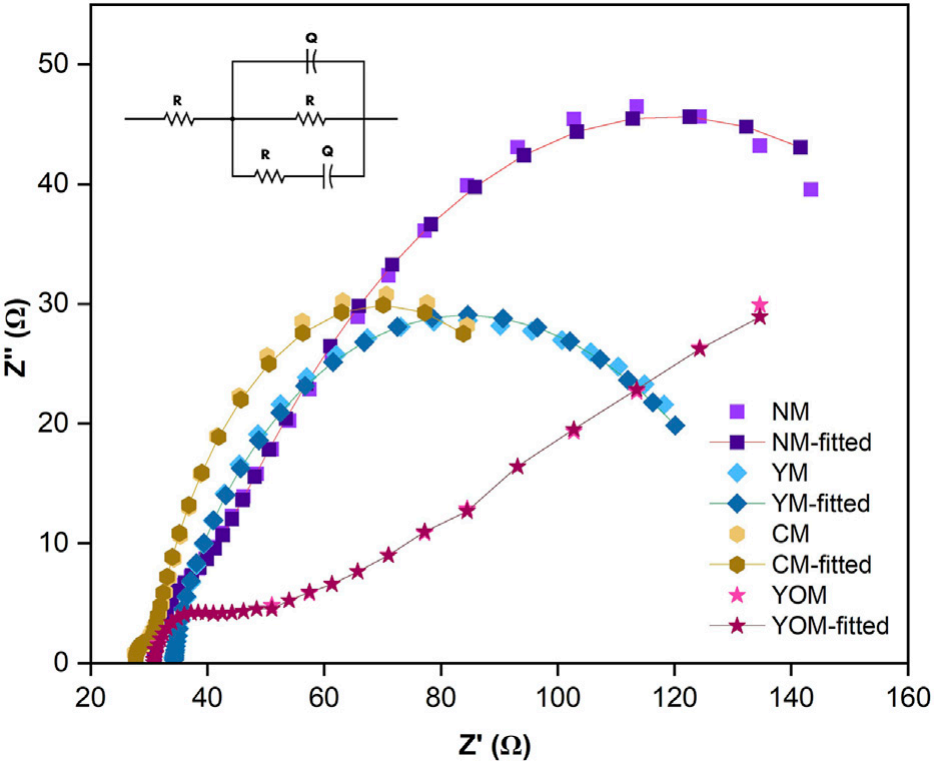
Nyquist plot for UC-loaded MoS_2_ CE-based DSSCs.

**FIGURE 10:**
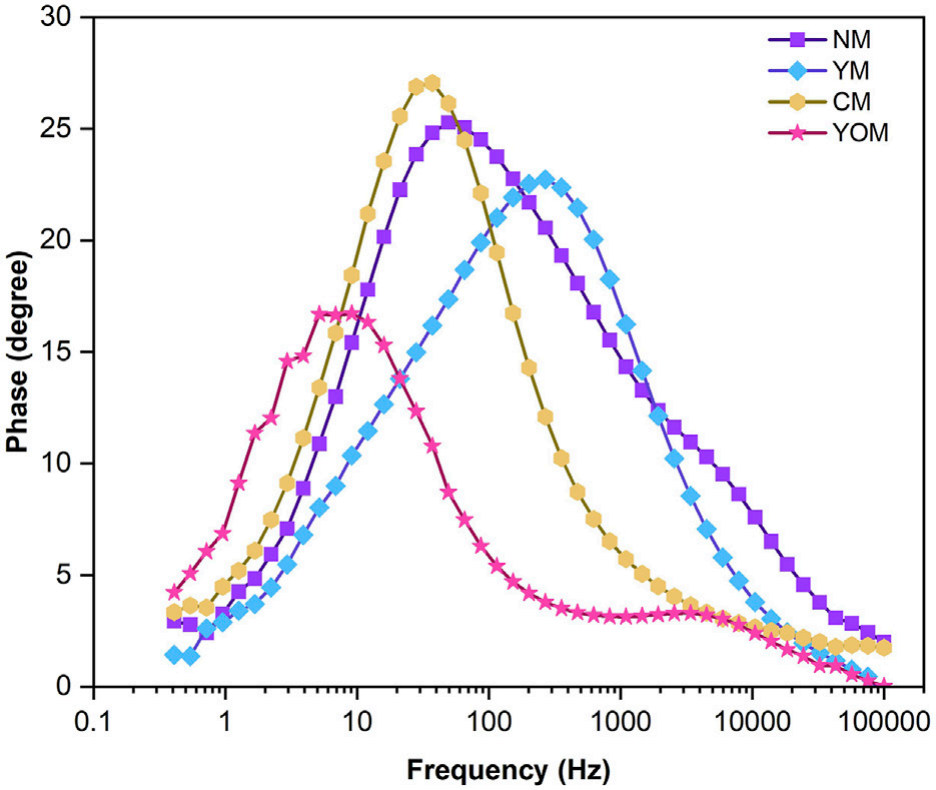
Bode-phase plot for UC-loaded MoS_2_ CE-based DSSCs.

The dynamics of the reactions and the internal resistance of DSSCs were analyzed by Electrochemical Impedance Spectroscopy (EIS). By fitting the data to an equivalent electrical circuit, the electrochemical parameters such as electron (τ_e_) lifetime, series (R_S_) and charge transfer (R_CT_) resistance were measured. The obtained Nyquist plot and Bode-Phase plot are shown in Figures 9, 10 respectively. The equivalent circuit represented in Figure 9 determines the internal resistances of the films by fitting the EIS spectrum data in the ZSimpWin software.

The semicircles observed at low and high-frequency ranges in the Nyquist plot correspond to the redox reaction happening in the CE/electrolyte interface and the electron transportation occurring at the photoanode/electrolyte interface (R_CT_). Table 1 displays the calculated R_S_ and R_CT_ values for the UC-loaded MoS_2_ CE-based DSSCs. Here in the Nyquist plot, the third semicircle is absent which is usually observed and this is because of the negligible separation of the TiO_2_ photoanode and UC-loaded MoS_2_ counter electrode and also the used iodine liquid electrolyte is a low viscous electrolyte. MoS_2_-based CEs generally have higher R_S_ values, which could be attributed to the minimal conductive value of the FTO substrate. Here, the Rs values of the UCs-loaded MoS_2_ counter electrodes are identical since Rs is correlated to the film on the substrate surface and the properties of each coated film obtained from the hydrothermal method on the FTO substrates are the same (Pujari et al., 2017). The YOM-based DSSC shows a lower R_S_ (30.31 Ω) value than other UCs (NM, YM& CM) loaded DSSCs, which suggests the higher conductivity of YOM-MoS_2_ CE. It is an indicator to assess the electronic conductivity and the low R_CT_ (1.76 Ω) value exhibited by YOM-based DSSC than other DSSC devices signifies a low electron recombination rate between CE and the electrolyte and also indicates the quicker mobility of electrons in the DSSC at the photoanode/electrolyte interface. This is mainly due to the in-depth exchange of hydrophilic act between the oxide UCs-loaded MoS_2_ (1T-2H phase) films and the FTO substrates (Yun et al., 2015). The hydrophilic nature of the mixed-phase of MoS_2_ shows good adhesion of the film onto the substrate. Also, the small R_CT_ value of oxide UCs might be owed to the films exposed with more catalytic active sites (1T-2H phase) favourable for the ion transportation in the I^−^/I^−3^ electrolyte. The bode phase plots of UC-loaded MoS_2_-based DSSCs are shown in Figure 10. The characteristic peak frequency (f_max_) for UC-loaded CEs was measured individually and the charge carrier lifetime (τ_e_) was determined by using the relation, τ_e_ = 1/(2πf_max_) and it was found to be 2.6 ms, 0.56 ms, 4.6 ms and 21.8 ms for NM, YM, CM, and YOM respectively. Here, the obtained results show that YOM-based DSSC has a longer electron lifetime which specifies the lower chance of recombination reactions to occur and thus exhibits excellent electrical conductivity and electrocatalytic activity than other DSSCs. Also, the existence of a large surface area with the porous nature of the YOM film prevents the electron-hole recombination reactions and possesses higher charge carrier concentration by having a large flat band potential and offers a prolonged lively pathway for faster electron transportation in the DSSC which necessarily enhance the J_SC_ of the DSSC (Sabari Girisun et al., 2023).

**TABLE 1:** Photovoltaic characteristics of UC-loaded MoS_2_ CE-based DSSCs.

Upconverter loaded MoS_2_		V_OC_ (V)	J_SC_ (mA/cm^2^)	FF	η %	R_S_ (Ω)	R_ct_ (Ω)	τ (ms)
Fluoride UCs	NM	0.687	7.29	0.51	3.02	30.94	8.81	2.63
	YM	0.685	5.49	0.51	2.28	30.70	13.39	0.56
Oxide UCs	CM	0.763	23.79	0.32	6.89	30.53	3.87	4.67
	YOM	0.792	24.96	0.31	7.19	30.31	1.76	21.82

**FIGURE 11:**
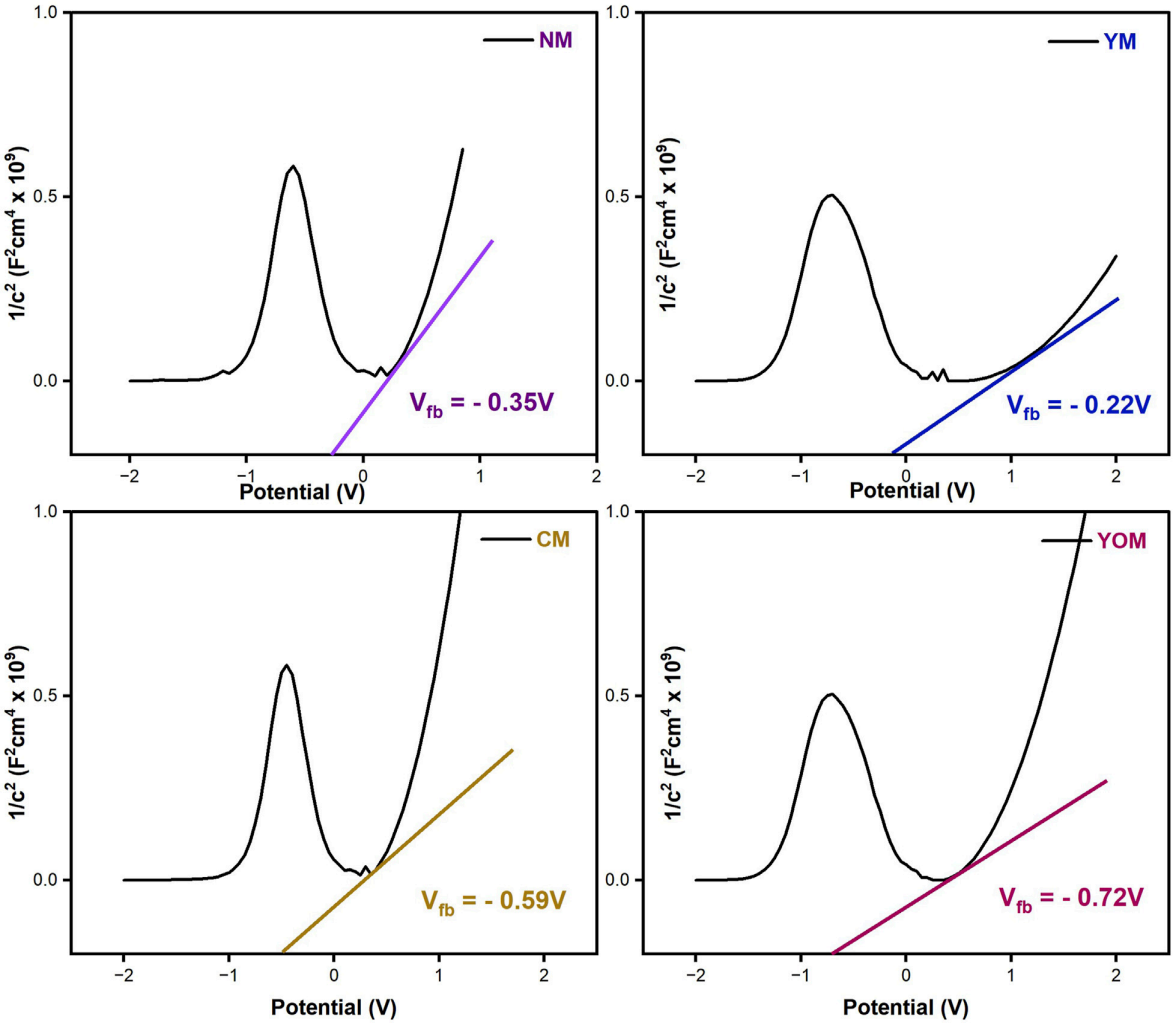
Mott-Schottky Curves for UC-loaded MoS_2_ films.

Additionally, Mott–Schottky (M–S) analysis was recorded with a 1 kHz frequency for all the UC-loaded MoS_2_ films to study the band potentials and the curves are shown in Figure 11. All the films exhibited a positive slope structure, which specifies that NM, YM, CM, and YOM films are n-type semiconductor electrodes in which electrons are the majority charge carriers. The flat band potentials (V_fb_) were calculated by taking slopes in the linear portion of the mott-Schottky curves. The estimated flat band potentials (V_fb_) for NM, YM, CM and YOM are −0.35 V, −0.22 V, −0.59 V and −0.72 V respectively. Generally, the flat band potential is directly associated to the charge carrier density in the semiconductor material. Here, it is evident from the plot, that YOM and CM oxide UCs show higher V_fb_ values than fluoride UCs which confirms the presence of a higher concentration of charge carriers in oxide UC films that arise mainly due to the presence of oxygen vacancies created by the oxide UCs in the MoS_2_ films. Among the chosen films, YOM reveals a larger flat band potential of −0.72 V which aids in improved electron conductivity and electrocatalytic activity and enhances the overall efficiency of the DSSC (Kumari and Kumar, 2023).

### 3.5 Photovoltaic performance of UC-loaded MoS_2_ CEs-based DSSC

**FIGURE 12:**
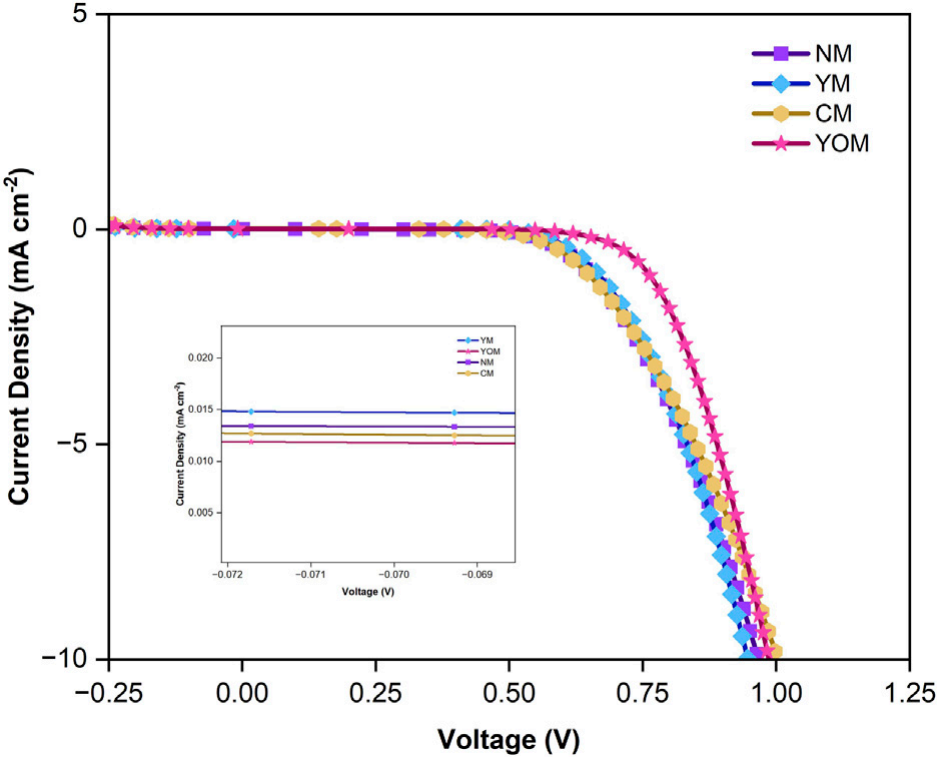
Dark J-V curve for UC-loaded MoS_2_ CE-based DSSCs.

**FIGURE 13:**
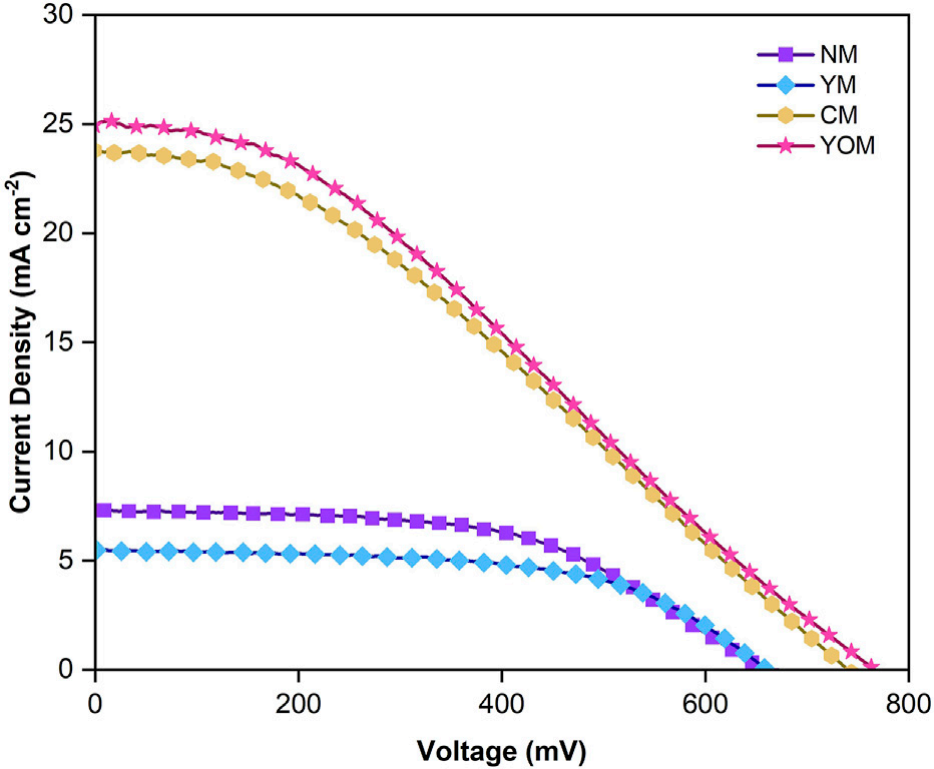
Light J-V curves for UC-loaded MoS_2_ CE-based DSSCs.

**FIGURE 14:**
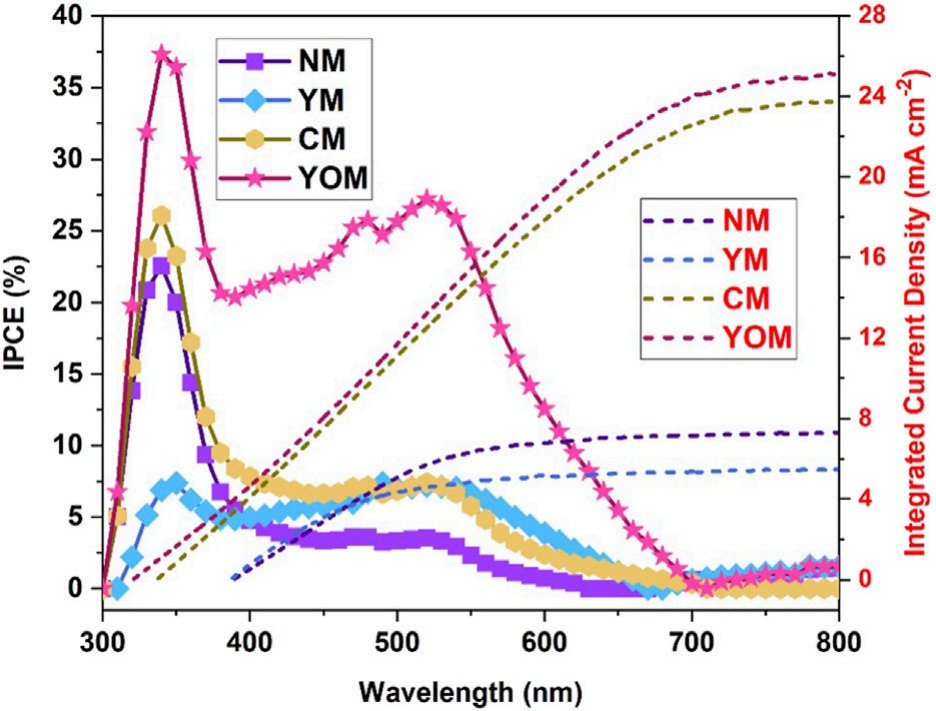
Integrated current density and IPCE curve for UC-loaded MoS_2_ CE-based DSSCs.

The I-V characteristics shown in Figure 12 for UC-loaded MoS_2_ CE-based DSSC under dark conditions are performed to study the process of current leakage, i.e., dark current due to the electron recombination in the DSSCs. The dark current for YOM (0.011 mA cm^−2^) and CM (0.012 mA cm^−2^) is smaller than for fluoride UCs-loaded DSSCs (NM & YM), as shown in the insert image in Figure 12. This indicates that the YOM and CM oxide UCs based DSSC can capably overturn the electron recombination process between the FTO substrate and the triiodide electrolyte and suggest a better photovoltaic performance under light condition by constraining the dark current and improving the short-circuit current. To analyze the impact of upconversion on photovoltaic performance of DSSCs, the UC-loaded MoS_2_ CE-based DSSCs fabricated with 0.25 cm^2^ active area were placed under Air Mass 1.5 illumination with input power of 85 mW cm^−2^. To facilitate upconversion-induced broadband absorption, an aluminium reflector beneath the CE of the DSSC to minimize the photon loss was placed. Figure 13 shows the light J-V curves of the DSSCs with UC-loaded MoS_2_ CE. The corresponding device characteristics (i.e., open-circuit (V_oc_) voltage, short-circuit current (J_sc_) density, fill factor ( $FF=\frac{\left(J_{M}\times V_{M}\right)}{\left(J_{SC}\times V_{OC}\right)}$ ) and photoconversion efficiency ( $PCE\;\left(\eta \;\%\right)=\frac{J_{SC}\times V_{OC}\times FF}{P_{in}}$ ) are tabulated in Table 1. From this measurement, the oxide UCs-loaded MoS_2_ CE-based DSSCs (CM = 6.89% & YOM = 7.19%) have higher PCE than fluoride UC-loaded MoS_2_ CE-based DSSCs (NM = 3.02% & YM = 2.28%). Table 2 summarizes the PV performance of similar upconverter nanoparticles loaded DSSC assemblies, MoS_2_ counter electrode-based DSSCs and Pt-based DSSCs prepared by various experimental conditions. The YOM-based DSSC has achieved a power conversion efficiency of 7.19% which was comparable with the Pt-based DSSCs signifying them to use as a substitute counter electrode for DSSC for broadband energy harvesting. The dropping of open circuit voltage and current density of fluoride UCs (NM and YM) loaded MoS_2_ CE based DSSCs denotes the ineffectual electron transportation in the DSSC referred to the occurrence of the high rate of electron recombination reactions, which is further confirmed by high R_CT_ values and low electron lifetime obtained from the EIS studies. Also, the existence of inadequate active edges in fluoride UCs to undergo electrocatalytic activity stands up as evidence to display lower performance than oxide UCs. Nevertheless, CM and YOM DSSCs demonstrate a steep increase in open circuit voltage (V_OC_) owing to the higher electrocatalytic action (low R_CT_) in reducing tri-iodide into iodide ions. This is mainly due to the decreased possibility of positive ions in the triiodide electrolyte circulating onto the TiO_2_ surface for recombination reactions. Here, V_OC_ of YOM (792.54 mV) and CM (763.24 mV) DSSCs display higher values than fluoride UCs-based DSSCs due to the lower recombination rate of electrons and the lesser number of positive ions in the triiodide electrolyte. This leads to sheltering additional injected photoelectrons from the dye and is crowded in the conduction level, directly increasing the quasi-fermi level of TiO_2_ and eventually enhancing the V_OC_ (Younas et al., 2020). The interlinked network of nanograins with porosity allows the external electrons to diffuse more proficiently between the UCs-loaded MoS_2_ electrodes and the triiodide electrolyte to improve the reduction reaction of the redox couple. Furthermore, the prolonged electron lifetime in oxide UCs-based DSSCs (CM = 4.6 ms and YOM = 21.8 ms) points to the lessening recombination reaction taking place in the DSSCs. The light-absorbing proficiency of the N719 dye, the suitable band configuration, and the ability of electron transfer from dye to TiO_2_ determines the current density (J_SC_) values of DSSCs and here increase in the J_SC_ of oxide UC (CM and YOM) loaded MoS_2_ CE based DSSCs can be associated with the prominent absorption of visible light due to the upconversion process enabled by upconverter nanoparticles and also the conductivity enhancement provided by the oxygen vacancy sites. Detailed characterization of the light-harvesting property of DSSCs is done by Incident Photon to Current Conversion Efficiency (IPCE) spectra which evolve from the N719 dye photo response in DSSCs to additionally validate the efficiency enhancement in YOM DSSC (Sabari Girisun et al., 2023). It can be seen that the IPCE responses are suggestively boosted in the whole visible region by introducing UCs, where the N719 dye molecules can be permitted to capture more photons, which can augment the PV performance of DSSCs. Also, a minor peak is spotted around the 750–800 nm region, i.e., in the NIR region due to the ytterbium ions present in the DSSCs (Li et al., 2014). The integrated current density (J_sc_) of DSSCs NM, YM, CM and YOM was determined from the IPCE graph and the curves are shown in Figure 14. The calculated integrated current density (J_sc_) of NM, YM, CM and YOM-based DSSCs were 7.32, 5.35, 23.58, and 25.02 mA/cm^2^, correspondingly. The acquired J_sc_ values from the IV plot remained in good consonance with the obtained J_sc_ values within the error limit and approved the precision of the photovoltaic measurements. Remarkably, the YOM DSSC exhibited higher IPCE response with 36.9% at the UV region and 27% at the visible region due to the strong upconversion luminescence intensity which may increase photon capturing and thus increase the J_SC_. Altogether successful incorporation of UC in CE to improve PCE of DSSC is demonstrated. In particular Y_2_O_3_: (Yb, Er) incorporated MoS_2_ CE based can be a potential component for the fabrication of Pt-free broadband light harvesting DSSCs.

**TABLE 2:** Photovoltaic characteristics of MoS_2_ CE-based DSSCs, UC-loaded DSSCs and Pt-based DSSCs.

Electrode	DSSC	V_OC_ (V)	J_SC_ (mA/cm^2^)	FF	η %	Reference
MoS_2_ Counter Electrode	MoS_2_- 16 nm	0.714	13.96	0.69	6.88	Jeong et al. (2019)
	MoS_2_- 30 min	0.730	15.92	0.61	7.14	Vikraman et al. (2019)
	MoS_2_- 24 h	0.755	14.77	0.49	6.42	Sabari Girisun et al. (2023)
	MoS_2_- Nanoflakes	0.730	10.10	0.69	5.1	Gopakumar et al. (2019)
	MoS_2_- Heat Sintered	0.705	13.01	0.65	5.96	Jeong et al. (2016)
	MoS_2_- Flexible Substrate	0.620	12.46	0.61	4.84	Gurulakshmi et al. (2020)
UC-loaded Counter Electrode	Er, Yb-FTO	0.744	18.44	-	7.30	Li et al. (2014)
UC-loaded Photoanode	BiYO_3_: Er/Yb	0.701	16.78	0.52	6.20	Dutta (2021)
	SnS- CeO_2_: Er/Yb	0.620	15.8	0.70	6.91	Lu et al. (2019)
	TiO_2_- LiYF_4_:Er/Yb	0.630	14.36	0.71	6.42	Ambapuram et al. (2020b)
Pt-based DSSCs	TiO_2_-Zn	0.670	15.89	0.54	5.74	Dubey et al. (2021)
	ZnO	0.707	14.47	0.67	6.93	Yang et al. (2016)
	P25 TiO_2_	0.770	11.17	0.68	6.89	Yoon et al. (2008)

## 4 Conclusion

In summary, the fluoride and oxide upconverter nanoparticles were successfully synthesized and introduced into the MoS_2_ counter electrodes, and the DSSCs were fabricated. Among the DSSCs, YOM-based DSSC Y_2_O_3_: (Yb, Er) shows high photovoltaic performance with 7.19% efficiency. The higher photocurrent generation of YOM-based DSSC is mainly due to the high electrocatalytic activity showed by low R_CT_ and R_S_ and the longer electron lifetime, which suggests the low electron recombination rate inside the DSSC. The absorption spectra demonstrate the wide-ranging absorption to NIR, i.e., up to 1,200 nm due to the inclusion of sensitizer (Yb^3+^ ions) in UCs and the PL spectra show that light emission is observed in the visible region by the non-radiative process of the emitter (Er^3+^ ions) where the N719 dye can captivate them for more electron excitation. These outcomes can deliver an effective understanding of the upconverter nanoparticles with the NIR light upconverting into visible light for high-performance dye-sensitized photovoltaic devices. It is intriguingly assured the UC-loaded MoS_2_ thin films can be used as an effective counter electrode for the possible realization of upconverter DSSC and this study provides an aspect for expanding the research to incorporating the UCs in DSSC for broadband absorption.

## Data Availability

The original contributions presented in the study are included in the article/supplementary material, further inquiries can be directed to the corresponding author.

## References

[B1] Ambapuram M. Maddala G. Simhachalam N. B. Sripada S. Kalvapalli S. Pedda V. S. Y. (2020a). Highly effective SnS composite counter electrode sandwiched bi-function CeO_2_: Er^3+^/Yb^3+^ assisted surface modified photoelectroded dye sensitized solar cell exceeds 9.5% efficiency. J. Sol. Energy. 207, 1158–1164. 10.1016/j.solener.2020.07.030

[B2] Ambapuram R. R. Maddala G. Godugunuru S. Yerva P. V. S. Mitty R. (2020b). Effective upconverter and light scattering dual function LiYF_4_: Er^3+^/Yb^3+^ assisted photoelectrode for high performance cosensitized dye sensitized solar cells. ACS Appl. Electron. Mater. 2 (4), 962–970. 10.1021/acsaelm.0c00014

[B3] Das G. S. Parida K. (2021). One step towards the 1T/2H-MoS_2_ mixed phase: a journey from synthesis to application. Mater. Chem. Front. 5 (5), 2143–2172. 10.1039/D0QM00802H

[B4] Dubey R. S. Jadkar S. R. Bhorde A. B. (2021). Synthesis and characterization of various doped TiO_2_ nanocrystals for dye-sensitized solar cells. ACS omega 5, 3470–3482. 10.1021/acsomega.0c01614 PMC7876674 33585733

[B5] Durairaj T. C. S. G. Sabari Girisun T. C. (2023). Demonstration of enhanced saturable absorption in upconverter integrated MoS_2_ heterostructure thin film using nanopulsed green laser. Phys. Status Solidi B Basic Res. 260 (11), 2300216. 10.1002/pssb.202300216

[B6] Dutta J. Rai V. K. Durai M. M. Thangavel R. (2019). Development of Y_2_O_3_: Ho^3+^/Yb^3+^ upconverting nanophosphors for enhancing solar cell efficiency of dye-sensitized solar cells. IEEE J. Photovolt. 9 (4), 1040–1045. 10.1109/JPHOTOV.2019.2912719

[B7] Dutta V. K. R. (2021). Upconverting BiYO_3_ nanophosphors in DSSCs applications. Opt. Laser Technol. 140, 107087. 10.1016/j.optlastec.2021.107087

[B8] El Assyry A. Rafiq I. Rbaa M. Derouiche A. Lakhrissi B. (2022). Optical and photovoltaic properties of new synthesized quinoxaline-2, 3 dione derivatives for efficient dye sensitized solar cell application. Trends Sci. 19 (19), 6173. 10.48048/tis.2022.6173

[B9] Fang X. Ma T. Guan G. Akiyama M. Kida T. Abe E. (2004). Effect of the thickness of the Pt film coated on a counter electrode on the performance of a dye-sensitized solar cell. J. Electroanal. Chem. 570 (2), 257–263. 10.1016/j.jelechem.2004.04.004

[B10] Ghaleghafi E. Rahmani M. B. Wei Z. H. (2021). Photoluminescence and UV photosensitivity of few-layered MoS_2_ nanosheets synthesized under different hydrothermal growth times. J. Mater. Sci. 56, 11749–11768. 10.1007/s10853-021-06083-x

[B11] Gopakumar G. Nair S. V. Shanmugam M. (2019). Hydrothermal processed heterogeneous MoS_2_ assisted charge transport in dye sensitized solar cells. Appl. Phys. 125 (12), 822. 10.1007/s00339-019-3126-3

[B12] Gurulakshmi A. M. Siddeswaramma G. Susmitha K. Subbaiah Y. P. V. Narayana T. Raghavender M. (2020). Electrodeposited MoS_2_ counter electrode for flexible dye sensitized solar cell module with ionic liquid assisted photoelectrode. J. Sol. Energy. 199, 447–452. 10.1016/j.solener.2020.02.047

[B13] Hussain S. F. S. Vikraman D. Mane R. S. Joo O. S. Naushad M. Jung J. (2015). High‐performance platinum‐free dye‐sensitized solar cells with molybdenum disulfide films as counter electrodes. ChemPhysChem 16 (18), 3959–3965. 10.1002/cphc.201500644 26472540

[B14] Jeong H. Kim J. Y. Koo B. Son H. J. Kim D. Ko M. J. (2016). Rapid sintering of MoS_2_ counter electrode using near-infrared pulsed laser for use in highly efficient dye-sensitized solar cells. J. Power Sources. 330, 104–110. 10.1016/j.jpowsour.2016.09.002

[B15] Jeong T. Ham S. Y. Koo B. Lee P. Min Y. S. Kim J. Y. (2019). Transparent 3 nm-thick MoS_2_ counter electrodes for bifacial dye-sensitized solar cells. J. Ind. Eng. Chem. 80, 106–111. 10.1016/j.jiec.2019.07.037

[B16] Kanjana W. M. Lunnoo T. Laokul P. Chaiya I. Ruammaitree A. Wongjom P. (2023). One-step hydrothermal synthesis and electrocatalytic properties of MoS_2_/activated carbon composite derived from shallots. J. Appl. Electrochem. 53 (12), 2311–2320. 10.1007/s10800-023-01921-z

[B17] Kumari R. Kumar R. (2023). Exploring the influence of temperature and time on the formation and properties of 3D flower-like MoS_2_ nanostructures synthesized via hydrothermal method. ECS J. Solid State Sci. Technol. 12 (9), 097004. 10.1149/2162-8777/acf8f1

[B18] Li L. Yang Y. Fan R. Chen S. Wang P. Yang B. (2014). Conductive upconversion Er, Yb-FTO nanoparticle coating to replace Pt as a low-cost and high-performance counter electrode for dye-sensitized solar cells. ACS Appl. Mater. Interfaces 6 (11), 8223–8229. 10.1021/am5009776 24810204

[B19] Lu X. Q. Zhang H. U. Li R. Zhang M. Guo M. (2019). Nb_2_O_5_ coating on the performance of flexible dye sensitized solar cell based on TiO_2_ nanoarrays/upconversion luminescence composite structure. Inorg. Mater. 34 (6), 590–598. 10.15541/jim20180406

[B20] Pujari R. B. Lokhande A. C. Shelke A. R. Kim J. H. Lokhande C. D. (2017). Chemically deposited nano grain composed MoS_2_ thin films for supercapacitor application. J. Colloid Interface Sci. 496, 1–7. 10.1016/j.jcis.2016.11.026 28209539

[B21] Sabari Girisun T. C. Durairaj M. Vijaya S. Anandan S. (2023). 1T and 2H phase molybdenum disulfide as a counter electrode for Pt free dye-sensitized solar cells. Mater. Sci. Eng. B 287, 116123. 10.1016/j.mseb.2022.116123

[B22] Sarkar A. Bera S. (2020). Chakraborty, CoNi_2_S_4_-reduced graphene oxide nanohybrid: an excellent counter electrode for Pt-free DSSC. J. Sol. Energy. 208, 139–149. 10.1016/j.solener.2020.07.075

[B23] Shan G. P. D. (2010). Near‐infrared sunlight harvesting in dye‐sensitized solar cells via the insertion of an upconverter‐TiO_2_ nanocomposite layer. J. Adv. Mater. 22 (39), 4373–4377. 10.1002/adma.201001816 20809511

[B24] Tadge R. S. Y. Vishwakarma P. K. Rai S. B. Chen T. M. Sapra S. Ray S. (2020). Enhanced photovoltaic performance of Y_2_O_3_: Ho^3+/^Yb^3+^ upconversion nanophosphor based DSSC and investigation of color tunability in Ho^3+^/Tm^3+^/Yb^3+^ tridoped Y_2_O_3_ . J. Alloys Compd. 821, 153230. 10.1016/j.jallcom.2019.153230

[B25] Tohluebaji N. Siri R. Muensit N. Putson C. Channuie P. Porrawatkul P. (2024). Hydrophobic and optical properties of P (VDF-HFP) nanofiber filled with nickel (II) chloride hexahydrate for dye-sensitized solar cells application. Trends Sci. 21 (9), 8762. 10.48048/tis.2024.8762

[B26] Vikraman A. A. A. Hussain S. Shrestha N. K. Jeong S. H. Jung J. Patil S. A. (2019). Design of WSe_2_/MoS_2_ heterostructures as the counter electrode to replace Pt for dye-sensitized solar cell. ACS Sustain. Chem. Eng. 7 (15), 13195–13205. 10.1021/acssuschemeng.9b02430

[B27] Vikraman S. A. P. Hussain S. Mengal N. Kim H. S. Jeong S. H. Jung J. (2018). Facile and cost-effective methodology to fabricate MoS_2_ counter electrode for efficient dye-sensitized solar cells. Dyes Pigm 151, 7–14. 10.1016/j.dyepig.2017.12.037

[B28] Wei W. Sun K. Hu Y. H. (2016). An efficient counter electrode material for dye-sensitized solar cells—flower-structured 1T metallic phase MoS_2_ . J. Mater. Chem. 4 (32), 12398–12401. 10.1039/C6TA04743B

[B29] Wilcoxon P. P. N. Samara G. A. (1996). Synthesis and optical properties of MoS_2_ nanoclusters. Mater Res. Soc. Symp. Proc. 452, 371. 10.1557/PROC-452-371

[B30] Yang Q. Duan J. Yang P. Tang Q. (2016). Counter electrodes from platinum alloy nanotube arrays with ZnO nanorod templates for dye-sensitized solar cells. Electrochim. Acta. 190, 648–654. 10.1016/j.electacta.2015.12.206

[B31] Yao N. Huang J. Fu K. Deng X. Ding M. Xu X. (2016). Rare earth ion doped phosphors for dye-sensitized solar cells applications. RSC Adv. 6 (21), 17546–17559. 10.1039/C5RA27033B

[B32] Yoon C. Vittal R. Lee J. Chae W.-S. Kim K.-J. (2008). Enhanced performance of a dye-sensitized solar cell with an electrodeposited-platinum counter electrode. Electrochim. Acta. 53 (6), 2890–2896. 10.1016/j.electacta.2007.10.074

[B33] Younas M. Baroud T. N. Gondal M. A. Dastageer M. A. Giannelis E. P. (2020). Highly efficient, cost-effective counter electrodes for dye-sensitized solar cells (DSSCs) augmented by highly mesoporous carbons. J. Power Sources. 468, 228359. 10.1016/j.jpowsour.2020.228359

[B34] Yu F. Shi Y. Shen X. Yao W. Han S. Ma J. (2018). Three-dimensional MoS_2_-nanosheet-based graphene/carbon nanotube aerogel as a Pt-free counter electrode for high-efficiency dye-sensitized solar cells. ACS Sustain. Chem. Eng. 6 (12), 17427–17434. 10.1021/acssuschemeng.8b03143

[B35] Yun S. Liu Y. Zhang T. Ahmad S. (2015). Recent advances in alternative counter electrode materials for Co-mediated dye-sensitized solar cells. Nanoscale 7 (28), 11877–11893. 10.1039/C5NR02433A 26132719

[B36] Zhao Y. Z. Yang X. Jiang X. Shen J. Li C. (2014). Plasmon-enhanced efficient dye-sensitized solar cells using core–shell-structured β-NaYF_4_:Yb, Er@SiO_2_@Au nanocomposites. J. Mater. Chem. 2 (39), 16523–16530. 10.1039/C4TA02230K

